# 24-Hour Blood Pressure Variability Via Ambulatory Monitoring and Risk for Probable Dementia in the SPRINT Trial

**DOI:** 10.14283/jpad.2024.35

**Published:** 2024-02-07

**Authors:** I. J. Sible, Daniel A. Nation

**Affiliations:** 1 Department of Psychology, University of Southern California, Los Angeles, CA 90089 USA; 2 Leonard Davis School of Gerontology, University of Southern California, Los Angeles, CA 90089 USA 3715 McClintock Ave,; 3 Zilkha Neurogenetic Institute, Department of Physiology and Neuroscience, University of Southern California, Los Angeles, CA 90089 USA

**Keywords:** Blood pressure variability, dementia, antihypertensives, ambulatory blood pressure monitoring

## Abstract

**Background:**

Blood pressure variability is an emerging risk factor for dementia, independent and oftentimes beyond mean blood pressure levels. Recent evidence from interventional cohorts with rigorously controlled mean blood pressure levels suggest blood pressure variability over months to years remains a risk for dementia, but no prior studies have investigated relationships with blood pressure variability over shorter time periods.

**Objectives:**

To investigate the potential effect of ambulatory blood pressure variability on the rate of cognitive outcomes under intensive vs standard blood pressure lowering.

**Design:**

Post hoc analysis of the randomized, controlled, open-label Systolic Blood Pressure Intervention Trial clinical trial.

**Setting:**

Multisite Systolic Blood Pressure Intervention Trial.

**Participants:**

793 participants at increased risk for cardiovascular disease and without history of dementia at study randomization.

**Intervention:**

Standard (<140 mmHg systolic blood pressure target) vs intensive (<120 mmHg systolic blood pressure target) lowering of mean blood pressure.

**Measurements:**

24-hour ambulatory blood pressure monitoring 27 months after treatment randomization (standard vs intensive) and follow-up cognitive testing. Intraindividual blood pressure variability was calculated as the average real variability over 24-hour, daytime, and nighttime periods. Participants were categorized into 3 adjudicated clinical outcomes: no cognitive impairment, mild cognitive impairment, probable dementia. Cox proportional hazards models examined the potential effect of ambulatory blood pressure variability on the rate of cognitive outcomes under intensive vs standard blood pressure lowering. Associations with mean blood pressure were also explored.

**Results:**

Higher systolic 24-hour blood pressure variability was associated with increased risk for probable dementia in the standard group (adjusted hazard ratio [HR]: 2.56 [95% CI 1.16, 5.62], p = 0.019) but not in the intensive group (HR: 0.54 [95% CI 0.24, 1.23], p = 0.141). Similar findings were observed with daytime systolic blood pressure variability but not nighttime blood pressure variability. Mean blood pressure was not associated with cognitive outcomes.

**Conclusions:**

Higher systolic 24-hour and daytime blood pressure variability via ambulatory monitoring is associated with risk for dementia under standard blood pressure treatment. Findings support prior evidence that blood pressure variability remains a risk for dementia despite strict control of mean blood pressure levels.

**Electronic Supplementary Material:**

Supplementary material is available in the online version of this article at 10.14283/jpad.2024.35.

## Introduction

**N**umerous observational studies suggest blood pressure (BP) variability (BPV), independent of mean BP, is an emerging risk factor for cognitive decline, dementia, and cerebrovascular disease (1–6). Once considered “noise” in BP management and research (7, 8) some studies now even indicate fluctuations in BP may have greater prognostic utility in predicting brain health outcomes than mean BP levels (1, 3). Mounting evidence for BPV as an independent risk factor for cognitive decline and dementia has fueled recent interest in studying the role of BPV in interventional cohorts, where the focus is typically on treating mean BP levels. By leveraging data from cohorts with rigorously controlled mean BP levels, such as the Systolic Blood Pressure Intervention Trial (SPRINT) clinical trial (9), several new studies have sought to identify the unique contribution of BPV to brain health outcomes. These retrospective studies suggest that higher BPV measured over a period of months to years remains a risk factor for cognitive decline (10), mild cognitive impairment (MCI), probable dementia (11, 12) and cerebral perfusion decline (13), despite strict control of mean BP levels. These findings are potentially important for BP management strategies relevant to brain health, given certain treatment approaches and classes of antihypertensive medications may be better than others at controlling both the mean and variability in BP levels (14–18).

In addition to variability over months to years, BP fluctuations can also be studied over shorter intervals, such as 24 hours, via ambulatory monitoring (7). Ambulatory monitoring captures the unique influence of the circadian rhythm on BP levels (e.g., morning surge, nighttime dip) that is strongly linked with cardiovascular health outcomes (e.g., risk for stroke and myocardial infarction) (19–21). This method has the added benefits of monitoring an individual’s BP in their everyday environment (vs clinic setting) and reducing the potential for white-coat hypertension or masked hypertension (7, 22). Several observational studies of BPV via ambulatory monitoring suggest links with cognitive decline (1, 23–27) but relationships with dementia risk remain understudied. Additionally, no prior studies have investigated whether BPV over 24 hours may relate to risk for dementia under specific antihypertensive strategies. Data linking BPV to cognitive decline under specific treatment conditions could inform BP therapeutics aimed at improving brain health, especially those planning to use emerging wearable BP technologies. To investigate this possibility, we conducted a post hoc analysis of the SPRINT trial to examine whether 24-hour BPV via ambulatory monitoring is related to dementia risk based on antihypertensive treatment type.

## Methods

### Participants

Data were obtained from the SPRINT trial, a publicly available deidentified dataset from the National Heart, Lung, and Blood Institute that has been described in detailed elsewhere (9, 28). The present investigation was a post hoc analysis of this data. SPRINT was a multicenter randomized, controlled study cohort trial in the United States and Puerto Rico conducted between November 2010 and March 2013 investigating whether intensive BP lowering could reduce cardiovascular risk when compared to standard BP treatment. Participants were recruited from the local community and a variety of clinical settings, such as primary care, nephrology, and geriatrics. At screening, participants were ≥ 50 years old, hypertensive (systolic BP 130 mmHg – 180 mmHg), and at risk for cardiovascular disease (≥1 of the following risk factors: history of cardiovascular disease, chronic kidney disease [estimated glomerular filtration rate < 60 mL/min per 1.73 m^2^], 10-year Framingham cardiovascular disease risk ≥ 15%, ≥ 75 years of age). Participants were excluded for history of stroke, diabetes, or heart failure, residing in a nursing home, diagnosis of dementia based on medical record review, or receiving medication primarily used to treat dementia. Participants were randomized 1:1 to either standard treatment (<140 mmHg systolic BP target) or intensive treatment (<120 mmHg systolic BP target). SPRINT was approved by an Institutional Review Board at each site. All participants provided their informed consent before treatment randomization.

### Measures

#### BP assessment

As previously reported (21), a subset of SPRINT participants (897/9361) underwent 24-hour ambulatory BP monitoring using SpaceLabs Medical Model 90207 monitors and standard collection protocol (29–32) within 3 weeks of the 27-month follow-up visit. The primary aim of the ambulatory monitoring ancillary study was to evaluate the effect of clinic-based antihypertensive therapies on ambulatory BP (21). During this 24-hour period, BP was recorded every 30 minutes from the participant’s non-dominant arm and the readings were not displayed. Recordings with <14 BP readings between 6:00 AM and 12:00 midnight and <6 readings between 12:00 midnight and 6:00 AM were excluded (21, 29, 32). Consistent with other ambulatory BP studies and to reduce the influence of wake/sleep transitions on BP (21, 33) daytime BPV was calculated from BP readings collected between 9:00 AM and 9:00 PM and nighttime BPV was calculated from BP readings collected between 1:00 AM and 6:00 AM. Intraindividual BPV was calculated as the average real variability (ARV) over the 24-hour period, daytime period, and nighttime period. Identical analyses using the standard deviation (SD), coefficient of variation [CV; 100 x SD/mean], and variability independent of mean (VIM) (34) of BPV are reported in the Supplementary Materials. Pulse pressure (systolic BP – diastolic BP) variability (ARV) was also calculated.

#### Cognitive assessment

Participants underwent cognitive testing at study baseline and every 2 years during the planned 4-year follow-up, and study closeout if it was >1 year after the planned 4-year follow-up (9). The cognitive battery included: Montreal Cognitive Assessment, Logical Memory I and II, Digit Symbol Coding, Hopkins Verbal Learning Test – Revised, Modified Rey-Osterrieth Complex Figure, 15-item Boston Naming Test, Category Fluency – Animals, Trail Making Test parts A and B, and Digit Span. As previously described (35), clinicians masked to treatment group classified participants into one of three adjudicated clinical outcomes based on cognitive test scores and other health information (mood, sleep, functional abilities, medications, hospitalizations, informant report of participant functional status): no cognitive impairment, MCI, or probable dementia. Standardized diagnostic criteria for MCI (36) and probable dementia (37) were used, as previously reported (38).

#### Data availability statement

All data are available through the SPRINT group.

### Statistical analysis

We used Cox proportional hazards models to examine the potential effects of ambulatory BPV (24-hour, daytime, nighttime ARV) on the rate of cognitive outcomes under intensive vs standard BP lowering, with adjustment for age, sex, education, race/ethnicity, and mean BP over the same 24-hour/daytime/nighttime period. Results using the SD, CV, and VIM of systolic BPV are reported in the Supplementary Materials. We focused our analyses on systolic BP given the focus of lowering systolic BP in the SPRINT trial (9), consistent with other BPV studies using this dataset (10, 11, 13, 39). However, analyses with diastolic BPV are reported in the Supplementary Materials. We also examined associations with 24-hour, daytime, and nighttime mean BP in order to directly compare potential effects with BPV. Exploratory analyses included 1) associations with pulse pressure variability; 2) interactions with sex; and 3) interactions with race (prespecified SPRINT subgroup Black vs non-Black (9, 40)) (Supplementary Materials). Sensitivity analyses additionally controlled for history of atrial fibrillation. The no cognitive impairment group was set as the reference group among the three adjudicated clinical outcomes. Participants classified with an adjudicated clinical outcome before undergoing ambulatory BP monitoring were excluded in the present analysis (n = 99). All analyses were 2-tailed with significance set at p < .05 and were carried out in R (41).

## Results

793 participants (n = 406 in the intensive group; n = 387 in the standard group) underwent ambulatory BP monitoring mean 27.6 (0.7 SD) months after treatment randomization (Table 1). The median (range) follow-up time after treatment randomization was 1957.5 (1070 – 2570) days for participants in the intensive group and 1948 (1056 – 2580) days for participants in the standard group. In the intensive group, 2/406 participants developed probable dementia and 7/406 participants developed MCI during follow-up. In the standard group, 6/387 participants developed probable dementia and 6/387 participants developed MCI during follow-up. As shown in Table 1, compared to participants in the standard group, participants in the intensive group had lower 24-hour mean BP, 24-hour BPV, daytime mean BP, daytime BPV, and nighttime mean BP (p’s = <0.001 – 0.014). The treatment groups did not significantly differ in nighttime BPV (p’s = 0.06 – 0.114).

**Table 1:** Baseline clinical and demographic information

	**Intensive (n = 406)**	**Standard (n = 387)**	**F or x2**	**p-value**
Age (years)	71.0 (9.1)	71.0 (9.6)	0.001	0.970
Sex (n, % female)	122 (30.1%)	112 (28.9%)	0.07	0.792
Race/ethnicity (n, %)			0.001	0.979
Black	109 (26.9%)	106 (27.4%)		
Hispanic	13 (3.2%)	7 (1.8%)		
White	272 (67.0%)	266 (68.7%)		
Other	12 (3.0%)	8 (2.1%)		
Education (n, %)			0.64	0.726
Less than college/other	230 (56.7%)	216 (55.8%)		
College	64 (15.8%)	69 (17.8%)		
Graduate school	112 (27.6%)	102 (26.4%)		
BMI (kg/m2)	29.2 (7.1)	29.0 (7.0)	.18	0.668
FRS 10-year risk score	20.2 (11.1)	20.1 (10.4)	.05	0.822
Medical history (n, %)				
Cardiovascular disease	77 (19.0%)	84 (21.7%)	.76	0.384
Hypertension	382 (94.1%)	357 (92.3%)	.79	0.375
Medication use (n, %)				
Antihypertensive agents	371 (91.4%)	350 (90.4%)	.11	0.736
No. antihypertensive agents used (median, IQR)	2 (2)	2 (2)	.96	0.326
24-hour systolic BP (mmHg)				
Mean	122.3 (11.6)	134.5 (11.8)	216.9	<0.001
ARV	9.8 (2.2)	10.4 (2.2)	10.87	0.001
Count (median, IQR)*	45 (6)	46 (5.5)	45.38	0.258
24-hour diastolic BP (mmHg)				
Mean	68.9 (7.8)	75.2 (10.1)	95.22	<0.001
ARV	7.3 (1.6)	7.8 (1.8)	14.73	<0.001
Daytime systolic BP (mmHg)				
Mean	126.23 (11.8)	139.3 (12.7)	224.8	<0.001
ARV	10.0 (2.9)	10.5 (2.8)	6.1	0.014
Count (median, IQR)*	22 (4)	23 (4)	30.2	0.507
Daytime diastolic BP (mmHg)				
Mean	72.2 (8.3)	79.1 (10.9)	99.32	<0.001
ARV	7.3 (2.3)	7.8 (2.5)	9.88	0.002
Nighttime systolic BP (mmHg)				
Mean	115.1 (14.0)	126.0 (14.7)	114.9	<0.001
ARV	9.3 (3.7)	9.8 (3.8)	3.55	0.06
Count (median, IQR)*	10 (0)	10 (0)	5.77	0.834
Nighttime diastolic BP (mmHg)				
Mean	63.4 (9.2)	68.9 (10.8)	59.43	<0.001
ARV	7.2 (2.8)	7.5 (3.0)	2.5	0.114

Means and SDs shown unless otherwise indicated; Bolded items indicate significant difference between treatment groups; *Number of valid BP readings over specified time period; Abbreviations: ARV = average real variability; BP = blood pressure; BMI = body mass index; FRS = Framingham Risk Score

### BPV

**Figure 1:**
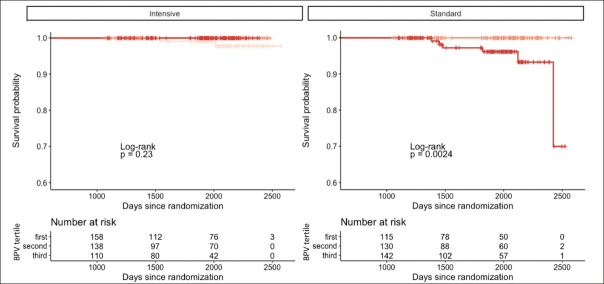
Higher 24-hour systolic BPV via ambulatory monitoring is associated with increased risk for probable dementia in the standard treatment group Kaplan-Meier curves showing event rates for probable dementia after stratification by treatment group.

As shown in Table 2 and Figure 1, higher 24-hour systolic BPV was associated with increased risk for probable dementia in the standard group (adjusted hazard ratio [HR]: 2.56 [95% CI 1.16, 5.62], p = 0.019) but not in the intensive group (HR: 0.54 [95% CI 0.24, 1.23], p = 0.141). Similar findings were observed with daytime systolic BPV (standard group HR: 1.53 [95% CI 1.05, 2.23], p = 0.026 vs intensive group HR: 0.58 [95% CI 0.29, 1.15], p = 0.116). Nighttime systolic BPV was not significantly associated with risk for probable dementia in either treatment group (p’s = 0.684 – 0.745). No systolic BPV metric (24-hour, daytime, nighttime) was related to risk for MCI (p’s = 0.074 – 0.551). Findings using the SD, CV, and VIM indices of BPV were consistent in direction, but were no longer significant (p’s = .065 – .072) (Supplementary Table 1). Diastolic BPV was not significantly associated with cognitive outcomes (Supplementary Table 2).

**Table 2:** Model estimates (adjusted hazard ratios [95% CI]) of ambulatory systolic BPV predicting cognitive outcomes

**HR (95% CI)**				
	**Intensive (n = 406)**	**p-value**	**Standard (n = 387)**	**p-value**
Probable dementia				
24-hour BPV	0.54 [0.24, 1.23]	0.141	2.56 [1.16, 5.62]	0.019
Daytime BPV	0.58 [0.29, 1.15]	0.116	1.53 [1.05, 2.23]	0.026
Nighttime BPV	0.94 [0.68, 1.29]	0.684	0.96 [0.75, 1.23]	0.745
MCI				
24-hour BPV	1.13 [0.82, 1.56]	0.462	0.83 [0.53, 1.29]	0.407
Daytime BPV	1.10 [0.87, 1.39]	0.417	0.90 [0.65, 1.25]	0.540
Nighttime BPV	0.93 [0.72, 1.19]	0.551	0.75 [0.55, 1.03]	0.074

Beta (β) and 95% confidence intervals shown unless otherwise indicated; Bolded items indicate ambulatory systolic BPV is significantly associated with risk for specified cognitive outcome; Models adjusted for age, sex, education, race/ethnicity, and mean systolic BP over the same 24-hour/daytime/nighttime period; Abbreviations: HR = hazard ratio; BPV = blood pressure variability; BP = blood pressure; MCI = mild cognitive impairment

### Mean BP

As shown in Table 3, mean systolic BP over any time period (24-hour, daytime, nighttime) was not associated with risk for probable dementia or MCI in either treatment group (p’s = 0.116 – 630).

**Table 3:** Model estimates (adjusted hazard ratios [95% CI]) of ambulatory systolic mean BP predicting cognitive outcomes

**HR (95% CI)**				
	**Intensive (n = 406)**	**p-value**	**Standard (n = 387)**	**p-value**
Probable dementia				
24-hour mean BP	1.08 [0.96, 1.21]	0.190	0.98 [0.93, 1.04]	0.523
Daytime mean BP	1.08 [0.97, 1.21]	0.180	0.97 [0.92, 1.02]	0.242
Nighttime mean BP	1.07 [0.97, 1.18]	0.183	1.01 [0.96, 1.07]	0.630
MCI				
24-hour mean BP	0.95 [0.88, 1.01]	0.118	1.04 [0.97, 1.11]	0.261
Daytime mean BP	0.97 [0.91, 1.04]	0.365	1.03 [0.97, 1.10]	0.334
Nighttime mean BP	0.95 [0.90, 1.01]	0.116	1.02 [0.96, 1.07]	0.569

Beta (β) and 95% confidence intervals shown unless otherwise indicated; Models adjusted for age, sex, education, and race/ethnicity; Abbreviations: HR = hazard ratio; BP = blood pressure; MCI = mild cognitive impairment

### Exploratory analyses

#### Pulse pressure variability

Higher daytime pulse pressure variability was associated with increased risk for probable dementia in the standard group (HR: 1.64 [95% CI 1.05, 2.56], p = 0.029) but not in the intensive group (HR: 0.98 [95% CI 0.66, 1.46], p = 0.931) (Supplementary Table 3). 24-hour and nighttime pulse pressure variability were not significantly related to risk for probable dementia in either treatment group (p’s = 0.055 – 0.976). Pulse pressure variability was not significantly associated with risk for MCI under intensive (p’s = 0.528 – 0.930) or standard (p’s = 0.088 – 0.856) treatment.

#### Interactions with sex and race

Interactions between BPV and 1) sex and 2) race on cognitive outcomes were all non-significant (p’s = 0.130 – 0.999) (Supplementary Table 4).

#### Sensitivity analyses

Higher 24-hour and daytime systolic BPV remained significantly associated with increased risk for probable dementia in the standard group after additionally controlling for history of atrial fibrillation (24-hour HR: 3.50 [95% CI 1.02, 12.00], p = 0.046; daytime HR: 1.52 [95% CI 1.04, 2.21], p = 0.029).

## Discussion

The present study was the first to examine ambulatory BPV and cognitive outcomes in the SPRINT dataset. In this post hoc analysis of the SPRINT trial, higher systolic BPV over 24 hours remains a risk factor for probable dementia despite strictly controlled mean BP levels, particularly in the context of standard BP lowering treatment. Additionally, daytime systolic BPV, but not nighttime BPV, was reduced in the intensive treatment group and was predictive of probable dementia in the standard treatment group. The present findings add information about clinical diagnosis to previous observational studies linking BPV via ambulatory monitoring to cognitive decline typically measured with single tests of cognition (1, 23–27). Additionally, the current study used data from a cohort with rigorously controlled mean BP levels, which enabled us to assess the contribution of BPV vs mean BP to dementia risk at a level not typically available in observational cohorts. Findings are consistent with prior observational (1) and interventional (11) work examining BPV over months to years and offer new evidence that BP fluctuations captured over 24 hours via ambulatory monitoring may also be an emerging vascular risk factor associated with cognitive impairment, cognitive decline, and dementia. More studies using this relatively newer method to measure BPV in other interventional cohorts are warranted to replicate these findings.

Associations between ambulatory BPV and dementia risk were observed only in the standard treatment group. It has been hypothesized that, compared to standard lowering of mean BP, intensive lowering mitigates the effects of BPV that are strongly linked with dementia risk in observational cohorts with varied BP control (1). However, other SPRINT studies on BPV measured over a period of months to years have found associations with cognitive outcomes in one or both treatment groups (10–12). Additionally, these SPRINT studies reported links with MCI (11, 12). Although the current study on ambulatory BPV did not identify an association with MCI, the hazard ratio for probable dementia under standard treatment in the current study of 24-hour ambulatory BPV (2.56) was greater than the hazard ratio (2.4) in a previous SPRINT study of BPV over years (12). 24-hour and daytime BPV was related to dementia risk in the standard treatment group, whereas nighttime BPV was not associated with dementia risk in either group. There are few observational studies on ambulatory BPV and cognitive impairment/decline (1, 42), and only one that directly compares 24-hour vs daytime vs nighttime BPV (25). Our findings with daytime BPV and not nighttime BPV are consistent with the study by McDonald et al., on relationships with cognitive decline. BP levels typically undergo dramatic changes during sleep (e.g., nighttime dip, morning surge) due to the influence of the circadian rhythm (7). It is therefore interesting that the associations with cognition in the present study and the study by McDonald et al., were different between daytime and nighttime BPV.

Despite a growing literature linking BPV to poor cerebrovascular health and dementia (3, 5, 6), the causes and consequences of BP fluctuations are still under investigation. Some have hypothesized that large variations in BP, regardless of mean BP levels or the time period of variation measured, may pose a risk to the health and function of the cerebral arteries by way of mechanical injury to the cerebrovascular compartments (5–7, 43), or triggering transcriptomic changes in endothelial cells through mechanoreceptors on their surface (44) that support neurovascular unit functioning (45). Several studies support this possibility and suggest that higher BPV is associated with markers of cerebrovascular disease (5, 6, 46, 47) and dysfunction (48, 49), which may ultimately contribute to cognitive impairment, cognitive decline, and dementia risk (45, 50). The current analysis did not directly address the potentially moderating effect of cerebrovascular disease, but a recent SPRINT study indicates that higher BPV over months, even with strictly controlled mean BP, is associated with cerebral perfusion decline in brain regions critical to cognitive function and vulnerable to Alzheimer’s disease (13). Although SPRINT ascertained adjudicated clinical outcomes for MCI and probable dementia, neuropathological information was not collected to confirm underlying pathologies. Prior observational work suggests elevated BPV, via ambulatory monitoring or other methods, is associated with Alzheimer’s disease and/or vascular disease process confirmed with plasma (51) cerebral spinal fluid (52), and postmortem evaluation (46, 47), but no studies to date have evaluated these associations under specific treatment conditions. Importantly, SPRINT participants had high vascular risk, which could increase the likelihood of comorbid vascular disease and Alzheimer’s disease. Future work that can elucidate underlying mechanisms and pathologies may have therapeutic implications for targeted dementia prevention. It is also important to note that underlying mechanisms driving BP fluctuations over shorter intervals – such as 24-hours in the present study – may be different from those driving fluctuations over longer intervals. For example, short-term fluctuations may reflect processes including baroreflex sensitivity, circadian rhythm, and emotional factors, whereas BPV over longer time intervals may be more related to arterial stiffening and adherence to antihypertensive therapies (7). Of course, it is expected that some of these mechanisms are shared across time intervals of BPV, and more research will help pinpoint specific therapeutic targets. Much of the work linking BPV to dementia risk has relied on mid- to long-term BPV (1, 42). Recent SPRINT post hoc analyses using clinic-based BPV measured over longer time intervals reported links with dementia risk in both the standard and intensive treatment groups (11, 12), whereas the present study using 24-hour BPV via ambulatory monitoring only found associations in the standard treatment group. The SPRINT trial was designed to treat clinic-based BP, and it is possible that different underlying mechanisms for short- and long-term BPV may be involved. Understanding mechanisms linking short-term BPV to dementia risk could add additional pharmacological and non-pharmacological targets for dementia prevention.

Interestingly, studies of BPV and brain health, including the present investigation, consistently report null or much weaker associations with mean BP levels (1, 6, 47), despite it being the overwhelming focus in current BP treatment strategies. This highlights the possibility that controlling BPV may require distinct interventions beyond standard approaches to BP control. Some evidence suggests differential antihypertensive class effects on BPV and risk for stroke (14, 15) and other cardiovascular events (17), and a recent study indicated this difference may also relate to dementia risk (16). Specifically, calcium-channel blockers may be superior in lowering both the mean and variability in BP levels (14, 16–18, 53). This finding is potentially important for translating BPV research into clinical practice since calcium-channel blockers are often recommended as firstline antihypertensives (18). Certainly, more research on class effects is needed, but this accumulating evidence has generated considerable discussion on how best to treat BPV (in addition to treating mean BP levels) and is in line with the current interest in precision-medicine approaches to care.

Rather than waiting months to years to accumulate BP recordings to calculate BPV, ambulatory BP monitoring may enable scientists and clinicians to estimate BPV in a matter of 24 hours and accelerate individualized treatment planning goals. Although ambulatory BP monitoring is not currently ubiquitous in clinical settings, more care sites and clinical trials may utilize this approach in the future (54). Ambulatory BP monitoring has the advantages of measuring BP in one’s everyday environment, which may reduce the likelihood of a white-coat effect or masked hypertension, and assessing the influence of circadian rhythms on BP. Recent advances in wearable BP technologies that make data collection more tolerable and accurate and the growing appetite for big data on various health metrics may also further promote this method. Additionally, the United States Preventative Services Task Force and the American College of Cardiology/American Heart Association made a grade A recommendation to use ambulatory BP monitoring to confirm a diagnosis of hypertension determined through standard office measurement, before initiating BP treatment (55–57). The present study captured 24-hour BPV via ambulatory monitoring well after initiating either intensive or standard lowering of mean BP - an average of 27 months after treatment randomization – and associations with dementia risk were still observed in a trial that was terminated early for cardiovascular benefit finding (58). The impact of BP therapies on cognition may take much longer to emerge than cardiovascular outcomes (35) and considering how to manage BPV at the outset of treatment may also benefit brain health. Of course, the SPRINT trial was not specifically focused on BPV – or ambulatory BP monitoring or cognitive outcomes - but future clinical trials that are may be interested in these approach considerations.

Findings provide novel evidence of a relationship between ambulatory BPV and clinical diagnosis and add to prior work relating to impairment and decline in typically single tests of cognition (1). The study is further strengthened by the use of data from an interventional cohort with rigorously controlled mean BP levels in terms of treatment initiation, dosing, titration, and adherence. Furthermore, the sample size is much larger than existing observational studies on ambulatory BP monitoring and cognitive outcomes (1). Additionally, the present investigation calculated BPV from ambulatory BP monitoring over just 24 hours and findings are consistent with previous studies measuring BPV over longer time periods (1, 3). SPRINT participants were diverse in terms of race and ethnicity, level of education, and geographical location, which increases generalizability of findings and demonstrates the feasibility, accessibility, and utility of BPV via ambulatory monitoring as a vascular measure linked to dementia risk. There are several study limitations worth noting. The BPV field is still emerging and standardized methods are not yet fully established (54). Ambulatory BP monitoring is also a relatively newer method to capture BPV when compared to standard office measurement and technological advances will likely promote more widespread use. Despite this fact, numerous studies using various BP collection methods provide compelling evidence that BPV may contribute to dementia risk in ways that are distinct from traditionally studied mean BP levels. Interestingly, associations with variability in more well-studied pulse pressure were similar to findings with BPV, albeit less robust (e.g., HR = 2.56 for 24-hour BPV and HR = 1.53 for daytime BPV vs HR = 1.89 for 24-hour pulse pressure variability and HR = 1.64 for daytime pulse pressure variability). This further highlights the possibly unique role that fluctuations in BP may play in brain health. As previously noted, we did not assess potential antihypertensive class effects. Studies with larger samples adequately powered to do so may enhance our understanding of BP therapeutics aimed at improving brain health. Although SPRINT participants were well characterized in terms of vascular factors, less is known about the cohort’s neuropathological burden. BP intervention trials that obtain this type of information have the potential to elucidate growing links between vascular factors and dementia risk (45, 50, 59, 60). Finally, the current study is limited by the fact that it is a post hoc analysis of an intervention trial focused on lowering mean BP levels. BP clinical trials and therapies that can address both the mean and variability in BP levels are warranted and may have therapeutic potential for reducing dementia risk.

## Conclusions

Higher systolic 24-hour and daytime BPV via ambulatory monitoring is associated with risk for dementia under standard BP treatment. Findings add to prior observational and interventional work studying BPV using various methods and highlight that BPV remains a risk for cognitive decline and dementia despite strict control of mean BP levels.

### Electronic supplementary material

Supplementary material, approximately 28 KB.

## Notes

*Ethical standards:* Procedures followed were in accordance with the ethical standards of the responsible committee on human experimentation (institutional and national) and with the Helsinki Declaration of 1975, as revised in 2000.

## References

[1] de Heus RAA, Tzourio C, Lee EJL (2021). Association Between Blood Pressure Variability With Dementia and Cognitive Impairment: A Systematic Review and Meta-Analysis. Hypertension.

[2] Lattanzi S, Vernieri F, Silvestrini M (2018). Blood pressure variability and neurocognitive functioning. J Clin Hypertens.

[3] Rouch L, Cestac P, Sallerin B (2020). Visit-to-visit blood pressure variability is associated with cognitive decline and incident dementia: The S.AGES cohort. Hypertension.

[4] de Heus RAA, Olde Rikkert MGM, Tully PJ, Lawlor BA, Claassen JAHR (2019). Blood Pressure Variability and Progression of Clinical Alzheimer Disease. Hypertension.

[5] Tully PJ, Yano Y, Launer LJ (2020). Association Between Blood Pressure Variability and Cerebral Small-Vessel Disease: A Systematic Review and Meta-Analysis. J Am Heart Assoc.

[6] Ma Y, Song A, Viswanathan A (2020). Blood Pressure Variability and Cerebral Small Vessel Disease: A Systematic Review and Meta-Analysis of Population-Based Cohorts. Stroke.

[7] Parati G, Ochoa JE, Lombardi C, Bilo G (2013). Assessment and management of blood-pressure variability. Nat Rev Cardiol.

[8] Parati G, Stergiou GS, Dolan E, Bilo G (2018). Blood pressure variability: clinical relevance and application. J Clin Hypertens.

[9] Ambrosius WT, Sink KM, Foy CG (2014). The design and rationale of a multicenter clinical trial comparing two strategies for control of systolic blood pressure: The Systolic Blood Pressure Intervention Trial (SPRINT). Clin Trials.

[10] Sible IJ, Nation DA. Blood Pressure Variability and Cognitive Decline: A Post Hoc Analysis of the SPRINT MIND Trial. Am J Hypertens. November 2022:hpac128. doi:10.1093/ajh/hpac128 10.1093/ajh/hpac128 PMC10208742 36448621

[11] de Havenon A, Anadani M, Prabhakaran S, Wong K, Yaghi S, Rost N (2021). Increased Blood Pressure Variability and the Risk of Probable Dementia or Mild Cognitive Impairment: A Post Hoc Analysis of the SPRINT MIND Trial. J Am Heart Assoc.

[12] Guo H, Tan Y, Yao Z, Zhang Z, Yan J, Meng X. Effect of visit-to-visit blood pressure variability on mild cognitive impairment and probable dementia in hypertensive patients receiving standard and intensive blood pressure treatment. Front Cardiovasc Med. 2023;10. https://www.frontiersin.org/articles/10.3389/fcvm.2023.1166554. 10.3389/fcvm.2023.1166554 PMC10150011 37139135

[13] Sible IJ, Nation DA. Blood Pressure Variability and Cerebral Perfusion Decline: A Post Hoc Analysis of the SPRINT MIND Trial. J Am Heart Assoc. 2023;0(0):e029797. doi:10.1161/JAHA.123.029797 10.1161/JAHA.123.029797 PMC10356024 37301768

[14] Webb AJ, Fischer U, Mehta Z, Rothwell PM (2010). Effects of antihypertensive-drug class on interindividual variation in blood pressure and risk of stroke: A systematic review and meta-analysis. Lancet.

[15] Rothwell PM, Howard SC, Dolan E (2010). Effects of β blockers and calcium-channel blockers on within-individual variability in blood pressure and risk of stroke. Lancet Neurol.

[16] Mahinrad S, Bennett DA, Sorond FA, Gorelick PB. Blood pressure variability, dementia, and role of antihypertensive medications in older adults. Alzheimer’s Dement. 2023;n/a(n/a). doi:10.1002/alz.12935 10.1002/alz.12935 PMC10354219 36656086

[17] de Havenon A, Petersen N, Wolcott Z (2022). Effect of dihydropyridine calcium channel blockers on blood pressure variability in the SPRINT trial: a treatment effects approach. J Hypertens.

[18] Lee J-W, Choi E, Son J-W (2020). Comparison of Blood Pressure Variability Between Losartan and Amlodipine in Essential Hypertension (COMPAS-BPV). Am J Hypertens.

[19] Pena-Hernandez C, Nugent K, Tuncel M (2020). Twenty-Four-Hour Ambulatory Blood Pressure Monitoring. J Prim Care Community Health.

[20] Krakoff LR (2013). Ambulatory blood pressure improves prediction of cardiovascular risk: implications for better antihypertensive management. Curr Atheroscler Rep.

[21] Drawz PE, Pajewski NM, Bates JT (2017). Effect of Intensive Versus Standard Clinic-Based Hypertension Management on Ambulatory Blood Pressure: Results From the SPRINT (Systolic Blood Pressure Intervention Trial) Ambulatory Blood Pressure Study. Hypertens (Dallas, Tex 1979).

[22] Gavriilaki M, Anyfanti P, Mastrogiannis K (2023). Association between ambulatory blood pressure monitoring patterns with cognitive function and risk of dementia: a systematic review and meta-analysis. Aging Clin Exp Res.

[23] Yu JH, Kim REY, Park SY (2022). Night blood pressure variability, brain atrophy, and cognitive decline. Front Neurol.

[24] Li L, Wang W, Lian T, et al. The Influence of 24-h Ambulatory Blood Pressure on Cognitive Function and Neuropathological Biomarker in Patients With Alzheimer’s Disease. Front Aging Neurosci. 2022;14. https://www.frontiersin.org/articles/10.3389/fnagi.2022.909582. 10.3389/fnagi.2022.909582 PMC9257169 35813940

[25] McDonald C, Pearce MS, Kerr SRJ, Newton JL. Blood pressure variability and cognitive decline in older people: a 5-year longitudinal study. J Hypertens. 2017;35(1). https://journals.lww.com/jhypertension/Fulltext/2017/01000/Blood_pressure_variability_and_cognitive_decline.22.aspx. 10.1097/HJH.0000000000001120 27648719

[26] Yamaguchi Y, Wada M, Sato H (2014). Impact of ambulatory blood pressure variability on cerebral small vessel disease progression and cognitive decline in community-based elderly Japanese. Am J Hypertens.

[27] Chiu T-J, Yeh J-T, Huang C-J (2021). Blood pressure variability and cognitive dysfunction: A systematic review and meta-analysis of longitudinal cohort studies. J Clin Hypertens.

[28] Rapp SR, Gaussoin SA, Sachs BC (2020). Effects of intensive versus standard blood pressure control on domain-specific cognitive function: a substudy of the SPRINT randomised controlled trial. Lancet Neurol.

[29] Drawz PE, Alper AB, Anderson AH (2016). Masked Hypertension and Elevated Nighttime Blood Pressure in CKD: Prevalence and Association with Target Organ Damage. Clin J Am Soc Nephrol.

[30] O’Brien E, Asmar R, Beilin L (2003). European Society of Hypertension recommendations for conventional, ambulatory and home blood pressure measurement. J Hypertens.

[31] O’Brien E, Coats A, Owens P (2000). Use and interpretation of ambulatory blood pressure monitoring: recommendations of the British hypertension society. BMJ.

[32] Gabbai FB, Rahman M, Hu B (2012). Relationship between ambulatory BP and clinical outcomes in patients with hypertensive CKD. Clin J Am Soc Nephrol.

[33] Parati G, Stergiou G, O’Brien E (2014). European Society of Hypertension practice guidelines for ambulatory blood pressure monitoring. J Hypertens.

[34] Rothwell PM, Howard SC, Dolan E (2010). Prognostic significance of visit-to-visit variability, maximum systolic blood pressure, and episodic hypertension. Lancet.

[35] Williamson JD, Pajewski NM, Auchus AP (2019). Effect of Intensive vs Standard Blood Pressure Control on Probable Dementia. JAMA.

[36] Albert MS, DeKosky ST, Dickson D (2011). The diagnosis of mild cognitive impairment due to Alzheimer’s disease: Recommendations from the National Institute on Aging-Alzheimer’s Association workgroups on diagnostic guidelines for Alzheimer’s disease. Alzheimer’s Dement.

[37] McKhann GM, Knopman DS, Chertkow H (2011). The diagnosis of dementia due to Alzheimer’s disease: Recommendations from the National Institute on Aging-Alzheimer’s Association workgroups on diagnostic guidelines for Alzheimer’s disease. Alzheimer’s Dement.

[38] Williamson JD, Pajewski NM, Auchus AP (2019). Effect of Intensive vs Standard Blood Pressure Control on Probable Dementia. JAMA.

[39] Cheng Y, Li J, Ren X (2021). Visit-to-visit office blood pressure variability combined with Framingham risk score to predict all-cause mortality: A post hoc analysis of the systolic blood pressure intervention trial. J Clin Hypertens.

[40] Wright JT, Williamson JD, Whelton PK (2015). A Randomized Trial of Intensive versus Standard Blood-Pressure Control. N Engl J Med.

[41] R Core Team. R: A language and environment for statistical computing. 2020. https://www.r-project.org/.

[42] Sun F (2023). The impact of blood pressure variability on cognition: current limitations and new advances. J Hypertens.

[43] Nagai M, Dote K, Kato M (2017). Visit-to-visit blood pressure variability and Alzheimer’s disease: Links and risks. J Alzheimer’s Dis.

[44] Höcht C, Ueshiba H, Komine H (2013). Blood Pressure Variability: Prognostic Value and Therapeutic Implications. ISRN Hypertens.

[45] Zlokovic B V (2011). Neurovascular pathways to neurodegeneration in Alzheimer’s disease and other disorders. Nat Rev Neurosci.

[46] Sible IJ, Bangen KJ, Blanken AE, Ho JK, Nation DA. Antemortem Visit-To-Visit Blood Pressure Variability Predicts Cerebrovascular Lesion Burden in Autopsy-Confirmed Alzheimer’s Disease. Wharton W, ed. J Alzheimer’s Dis. 2021;83(1):65–75. doi:10.3233/JAD-210435 10.3233/JAD-210435 PMC8820262 34250941

[47] Ma Y, Blacker D, Viswanathan A (2021). Visit-to-visit blood pressure variability, neuropathology, and cognitive decline. Neurology.

[48] Sible IJ, Yew B, Dutt S (2022). Selective vulnerability of medial temporal regions to short-term blood pressure variability and cerebral hypoperfusion in older adults. Neuroimage: Reports.

[49] Sible IJ, Jang JY, Dutt S, et al. Older adults with higher blood pressure variability exhibit cerebrovascular reactivity deficits. Am J Hypertens. September 2022:hpac108. doi:10.1093/ajh/hpac108 10.1093/ajh/hpac108 PMC9793985 36149821

[50] Iadecola C (2004). Neurovascular regulation in the normal brain and in Alzheimer’s disease. Nat Rev Neurosci.

[51] Sible IJ, Yew B, Jang JY (2022). Blood pressure variability and plasma Alzheimer’s disease biomarkers in older adults. Sci Rep.

[52] Sible IJ, Nation DA (2020). Long-term blood pressure variability across the clinical and biomarker spectrum of Alzheimer’s disease. J Alzheimer’s Dis.

[53] Levi-Marpillat N, Macquin-Mavier I, Tropeano A-I, Parati G, Maison P (2014). Antihypertensive drug classes have different effects on short-term blood pressure variability in essential hypertension. Hypertens Res.

[54] Sheikh AB, Sobotka PA, Garg I (2023). Blood Pressure Variability in Clinical Practice: Past, Present and the Future. J Am Heart Assoc.

[55] Piper MA, Evans C V, Burda BU, Margolis KL, O’Connor E, Whitlock EP (2015). Diagnostic and predictive accuracy of blood pressure screening methods with consideration of rescreening intervals: a systematic review for the U.S. Preventive Services Task Force. Ann Intern Med.

[56] Siu AL (2015). Screening for high blood pressure in adults: U.S. Preventive Services Task Force recommendation statement. Ann Intern Med.

[57] Whelton PK, Carey RM, Aronow WS (2018). 2017 ACC/AHA/AAPA/ABC/ACPM/AGS/APhA/ASH/ASPC/NMA/PCNA Guideline for the Prevention, Detection, Evaluation, and Management of High Blood Pressure in Adults: A Report of the American College of Cardiology/American Heart Association Task Force on Clinical Pr. Hypertension.

[58] Wright JT, Williamson JD, Whelton PK (2015). A randomized trial of intensive versus standard blood-pressure control. N Engl J Med.

[59] Iadecola C, Yaffe K, Biller J (2016). Impact of hypertension on cognitive function: A scientific statement from the American Heart Association. Hypertension.

[60] Gorelick PB, Scuteri A, Black SE (2011). Vascular contributions to cognitive impairment and dementia: A statement for healthcare professionals from the American Heart Association/American Stroke Association. Stroke.

