# Supplementary material for: 24-Hour Blood Pressure Variability Via Ambulatory Monitoring and Risk for Probable Dementia in the SPRINT Trial
Source: J Prev Alzheimers Dis. 2024 Feb 7;11(3):684–92. doi: 10.14283/jpad.2024.35 (PMC11060998; doi:10.14283/jpad.2024.35)
Supplement: Supplementary file 1 — Supplementary material, approximately 28 KB. [file 42414_2024_35_MOESM1_ESM.docx]

**Supplementary Table 1.**

Model estimates (adjusted hazard ratios [95% CI]) of ambulatory systolic BPV (SD, CV, VIM) predicting cognitive outcomes.

| **HR (95% CI)** | | | | |
| --- | --- | --- | --- | --- |
|  | **Intensive (*n* = 406)** | ***p*-value** | **Standard (*n* = 387)** | ***p*-value** |
| Probable dementia |  |  |  |  |
| 24-hour BPV |  |  |  |  |
| SD | 0.75 [0.45, 1.25] | 0.265 | 1.27 [0.98, 1.63] | 0.067 |
| CV | 0.76 [0.41, 1.41] | 0.385 | 1.38 [0.98, 1.93] | 0.065 |
| VIM | 0.44 [0.09, 2.16] | 0.311 | 2.86 [0.92, 8.88] | 0.069 |
| Daytime BPV |  |  |  |  |
| SD | 0.57 [0.33, 0.98] | 0.042 | 1.29 [0.98, 1.70] | 0.068 |
| CV | 0.51 [0.22, 1.19] | 0.118 | 1.42 [0.97, 2.09] | 0.072 |
| VIM | 0.18 [0.03, 0.99] | 0.049 | 2.89 [0.93, 9.05] | 0.068 |
| Nighttime BPV |  |  |  |  |
| SD | 1.05 [0.76, 1.46] | 0.771 | 1.11 [0.88, 1.39] | 0.381 |
| CV | 1.07 [0.71, 1.62] | 0.732 | 1.15 [0.87, 1.53] | 0.333 |
| VIM | 1.40 [0.34, 5.67] | 0.640 | 1.68 [0.64, 4.39] | 0.293 |
| MCI |  |  |  |  |
| 24-hour BPV |  |  |  |  |
| SD | 1.13 [0.92, 1.40] | 0.242 | 0.97 [0.77, 1.22] | 0.778 |
| CV | 1.16 [0.91, 1.49] | 0.242 | 0.95 [0.69, 1.31] | 0.741 |
| VIM | 1.56 [0.74, 3.33] | 0.246 | 0.88 [0.36, 2.17] | 0.779 |
| Daytime BPV |  |  |  |  |
| SD | 1.04 [0.84, 1.29] | 0.691 | 0.94 [0.73, 1.22] | 0.653 |
| CV | 1.06 [0.82, 1.37] | 0.677 | 0.92 [0.64, 1.32] | 0.637 |
| VIM | 1.17 [0.58, 2.38] | 0.656 | 0.76 [0.30, 1.95] | 0.571 |
| Nighttime BPV |  |  |  |  |
| SD | 0.94 [0.73, 1.21] | 0.630 | 0.79 [0.59, 1.05] | 0.104 |
| CV | 0.94 [0.71, 1.24] | 0.653 | 0.72 [0.49, 1.05] | 0.087 |
| VIM | 0.82 [0.35, 1.93] | 0.642 | 0.44 [0.17, 1.12] | 0.084 |

Beta (ß) and 95% confidence intervals shown unless otherwise indicated.

Models adjusted for age, sex, education, race/ethnicity, and mean systolic BP over the same 24-hour/daytime/nighttime period.

Abbreviations: HR = hazard ratio; BPV = blood pressure variability; BP = blood pressure; MCI = mild cognitive impairment; SD = standard deviation; CV = coefficient of variation; VIM = variability independent of mean

**Supplementary Table 2.**

Model estimates (adjusted hazard ratios [95% CI]) of ambulatory diastolic BPV predicting cognitive outcomes.

| **HR (95% CI)** | | | | |
| --- | --- | --- | --- | --- |
|  | **Intensive (*n* = 406)** | ***p*-value** | **Standard (*n* = 387)** | ***p*-value** |
| Probable dementia |  |  |  |  |
| 24-hour BPV | 0.88 [0.35, 2.21] | 0.787 | 1.19 [0.71, 2.00] | 0.501 |
| Daytime BPV | 1.15 [0.56, 2.35] | 0.699 | 1.09 [0.79, 1.50] | 0.613 |
| Nighttime BPV | 0.99 [0.57, 1.73] | 0.976 | 0.89 [0.60, 1.35] | 0.592 |
| MCI |  |  |  |  |
| 24-hour BPV | 1.24 [0.80, 1.91] | 0.339 | 0.75 [0.43, 1.30] | 0.300 |
| Daytime BPV | 1.22 [0.89, 1.68] | 0.223 | 0.71 [0.44, 1.15] | 0.168 |
| Nighttime BPV | 1.07 [0.83, 1.37] | 0.604 | 0.89 [0.64, 1.25] | 0.503 |

Beta (ß) and 95% confidence intervals shown unless otherwise indicated.

Models adjusted for age, sex, education, race/ethnicity, and mean diastolic BP over the same 24-hour/daytime/nighttime period.

Abbreviations: HR = hazard ratio; BPV = blood pressure variability; BP = blood pressure; MCI = mild cognitive impairment

**Supplementary Table 3.**

Model estimates (adjusted hazard ratios [95% CI]) of ambulatory pulse pressure variability (ARV) predicting cognitive outcomes.

| **HR (95% CI)** | | | | |
| --- | --- | --- | --- | --- |
|  | **Intensive (*n* = 406)** | ***p*-value** | **Standard (*n* = 387)** | ***p*-value** |
| Probable dementia |  |  |  |  |
| 24-hour BPV | 0.92 [0.48, 1.77] | 0.797 | 1.89 [0.99, 3.61] | 0.055 |
| Daytime BPV | 0.98 [0.66, 1.46] | 0.931 | 1.64 [1.05, 2.56] | 0.029 |
| Nighttime BPV | 0.92 [0.55, 1.56] | 0.764 | 1.01 [0.74, 1.36] | 0.976 |
| MCI |  |  |  |  |
| 24-hour BPV | 0.98 [0.63, 1.50] | 0.910 | 0.56 [0.29, 1.09] | 0.088 |
| Daytime BPV | 0.99 [0.73, 1.34] | 0.930 | 0.97 [0.70, 1.35] | 0.856 |
| Nighttime BPV | 0.91 [0.67, 1.23] | 0.528 | 0.82 [0.59, 1.15] | 0.252 |

Beta (ß) and 95% confidence intervals shown unless otherwise indicated.

Models adjusted for age, sex, education, race/ethnicity, and mean pulse pressure over the same 24-hour/daytime/nighttime period.

Abbreviations: HR = hazard ratio; BPV = blood pressure variability; BP = blood pressure; MCI = mild cognitive impairment

**Supplementary Table 4.**

Model estimates (adjusted hazard ratios [95% CI]) of ambulatory systolic BPV interaction with 1) sex and 2) race (Black vs non-Black) on predicting cognitive outcomes

| **HR (95% CI)** | | | | |
| --- | --- | --- | --- | --- |
|  | **Intensive (*n* = 406)** | ***p*-value** | **Standard (*n* = 387)** | ***p*-value** |
| Probable dementia |  |  |  |  |
| 24-hour BPV |  |  |  |  |
| x Sex | 0.46 [0.00, 1.00] | 0.999 | 0.31 [0.04, 2.64] | 0.285 |
| x Race | 0.40 [0.00, 1.00] | 0.999 | 0.49 [0.00, 1.00] | 0.999 |
| Daytime BPV |  |  |  |  |
| x Sex | 0.60 [0.00, 1.00] | 0.999 | 0.99 [0.57, 1.72] | 0.976 |
| x Race | 0.36 [0.00, 1.00] | 0.999 | 1.93 [0.00, 1.00] | 0.999 |
| Nighttime BPV |  |  |  |  |
| x Sex | 0.88 [0.00, 1.00] | 0.999 | 0.73 [0.47, 1.14] | 0.167 |
| x Race | 0.89 [0.00, 1.00] | 0.999 | 0.76 [0.00, 1.00] | 0.999 |
| MCI |  |  |  |  |
| 24-hour BPV |  |  |  |  |
| x Sex | 1.09 [0.50, 2.41] | 0.822 | 0.65 [0.27, 1.54] | 0.324 |
| x Race | 1.69 [0.44, 6.52] | 0.448 | 2.58 [0.76, 8.82] | 0.130 |
| Daytime BPV |  |  |  |  |
| x Sex | 1.01 [0.62, 1.64] | 0.982 | 0.76 [0.39, 1.46] | 0.406 |
| x Race | 1.32 [0.51, 3.44] | 0.572 | 0.84 [0.42, 1.67] | 0.613 |
| Nighttime BPV |  |  |  |  |
| x Sex | 1.02 [0.57, 1.82] | 0.951 | 0.78 [0.45, 1.33] | 0.355 |
| x Race | 2.51 [0.59, 10.80] | 0.215 | 1.23 [0.62, 2.45] | 0.556 |

Beta (ß) and 95% confidence intervals shown unless otherwise indicated.

Models adjusted for age, sex, education, and mean BP over the same 24-hour/daytime/nighttime period.

Abbreviations: HR = hazard ratio; BPV = blood pressure variability; BP = blood pressure; MCI = mild cognitive impairment
